# An Etiological Model of Perfectionism

**DOI:** 10.1371/journal.pone.0094757

**Published:** 2014-05-01

**Authors:** Gayle K. Maloney, Sarah J. Egan, Robert T. Kane, Clare S. Rees

**Affiliations:** School of Psychology and Speech Pathology, Curtin University and Curtin Health Innovation Research Institute, Perth, Western Australia; Univ of Toledo, United States of America

## Abstract

**Objective:**

Perfectionism has been recognized as a transdiagnostic factor that is relevant to anxiety disorders, eating disorders and depression. Despite the importance of perfectionism in psychopathology to date there has been no empirical test of an etiological model of perfectionism.

**Method:**

The present study aimed to address the paucity of research on the etiology of perfectionism by developing and testing an etiological model using a sample of 311 clients seeking treatment.

**Results:**

Structural equation modeling showed a direct relationship between high Parental Expectations and Criticism, and Perfectionism. There was also an indirect relationship between Parental Bonding and Perfectionism that was mediated by core schemas of disconnection and rejection. Finally, it was found that Neuroticism had both an indirect relationship, which was mediated by core schemas, and a direct relationship with perfectionism.

**Conclusions:**

The study provided the first direct test of an etiological model of perfectionism to date. Clinical implications include investigating whether the inclusion of etiological factors in the understanding and treatment of perfectionism is effective.

## Introduction

There is extensive evidence that perfectionism is elevated in anxiety disorders, eating disorders and depression [1]. While there have been models developed to explain the maintenance of clinical perfectionism [2], [3], few researchers have developed models of etiology. Flett and colleagues [4] developed the only etiological model of perfectionism to date to explain the onset of self-oriented perfectionism (expecting perfection of oneself), socially-prescribed perfectionism (others expecting perfection of the individual) and other-oriented perfectionism (expecting others to be perfect) [5]. Flett et al. [4] proposed that perfectionism develops due to an interaction of parental, temperament and environmental factors. Despite this, the model remains untested. The aim of the present research was to survey the literature to identify the salient factors that have a consistent association with perfectionism, and then to configure them in a proposed etiological model.

Barlow's [6] model based on the co-ordination of triple vulnerabilities: generalized biological vulnerability, early life experiences and specific psychological vulnerabilities, was considered a useful guide for identifying factors to include in an etiological model. It is assumed that anxiety and other emotional disorders have a common genetic basis and it is the co-ordination of specific environmental factors that determine the development of a specific psychological construct, such as perfectionism. There is evidence of a biological vulnerability for perfectionism; for example [7], [8], which could not be directly tested in the present research. Pertinent environmental and cognitive factors, however, were identified following a review of the literature.

Parental bonding, as measured by the Affectionless Control subscale of the Parental Bonding Inventory [9], appears to be the most widely recognized etiological factor for perfectionism [10], [11]. There is also strong theoretical consensus that perfectionistic and demanding parenting contributes to the development of perfectionism, with parental expectations and criticism being included as subscales on the widely used Frost Multidimensional Perfectionism Scale (FMPS) [12]. A relationship between modeling of high parental expectations and perfectionism has been consistently found [13], [14]. Similarly, a consistent relationship has been found between parental criticism and perfectionism [15]. Therefore, parental expectations and criticism appear to play an important etiological role in the development of perfectionism.

The personality dimension of Neuroticism has consistently been found to have a strong positive association with perfectionism. Neuroticism, as measured by the five-factor model of personality, is positively associated with measures of perfectionism; for example: [16]–[20] and was therefore included in the proposed model.

Cognitive models; for example: [21] suggest the interaction of environmental factors and core schemas may mediate the development of certain beliefs, such as perfectionism. There is evidence for several core schemas mediating the relationship between parental bonding and eating disordered and depressive psychopathology [22]–[26]. Given the established link between perfectionism and psychopathology [1], it may be that core schemas play a similar mediating role between parental factors and perfectionism. However, no models of perfectionism have included core schemas as an etiological factor, and the inclusion of cognitive factors in models of perfectionism appears to be an area that warrants further research.

There has been a paucity of research examining core schemas and perfectionism directly. In the cognitive-behavioural model of the maintenance of clinical perfectionism, self-worth being dependent on achievement has been seen as the core maintaining factor [2], [3]. Some research has demonstrated the role of contingent self-worth in perfectionism [27], [28]. It could be argued, however, that contingent self-worth is only one of several potential core beliefs that should be examined. A study of eating pathology and core schemas conducted by Waller and colleagues [29] found a positive association between perfectionism, as measured by the EDI-2 [30], and several core schemas using the Young Schema Questionnaire (YSQ) [31]. The YSQ-Short Form (YSQ-SF) has also been used to measure core schemas in studies investigating their mediating role in depressive [25] and eating disorder samples [23], [24], [26]. Boone and colleagues [32] investigated the YSQ with 88 females with eating disorders and found that personal standards, concern over mistakes and doubts about actions on the Frost Multidimensional Perfectionism Scale [12] were significantly related to the schema domains of Disconnection, Other-Directedness and Overvigilance.

Furthermore, the YSQ-SF provides a measure of the schema domain of Disconnection and Rejection that is consistent with the underlying core schemas proposed in cognitive models; for example: [21] of Helplessness and Unlovability. This Disconnection and Rejection schema domain includes five core beliefs of Emotional Deprivation, Abandonment, Mistrust/Abuse, Defectiveness, and Social Undesirability/Isolation. Theorists have consistently proposed that Core beliefs pertaining to these themes are related to perfectionism [28], [2]. It would therefore be useful to examine Disconnection and Rejection schemas as one of the potential etiological factors for perfectionism.

**Figure 1:**
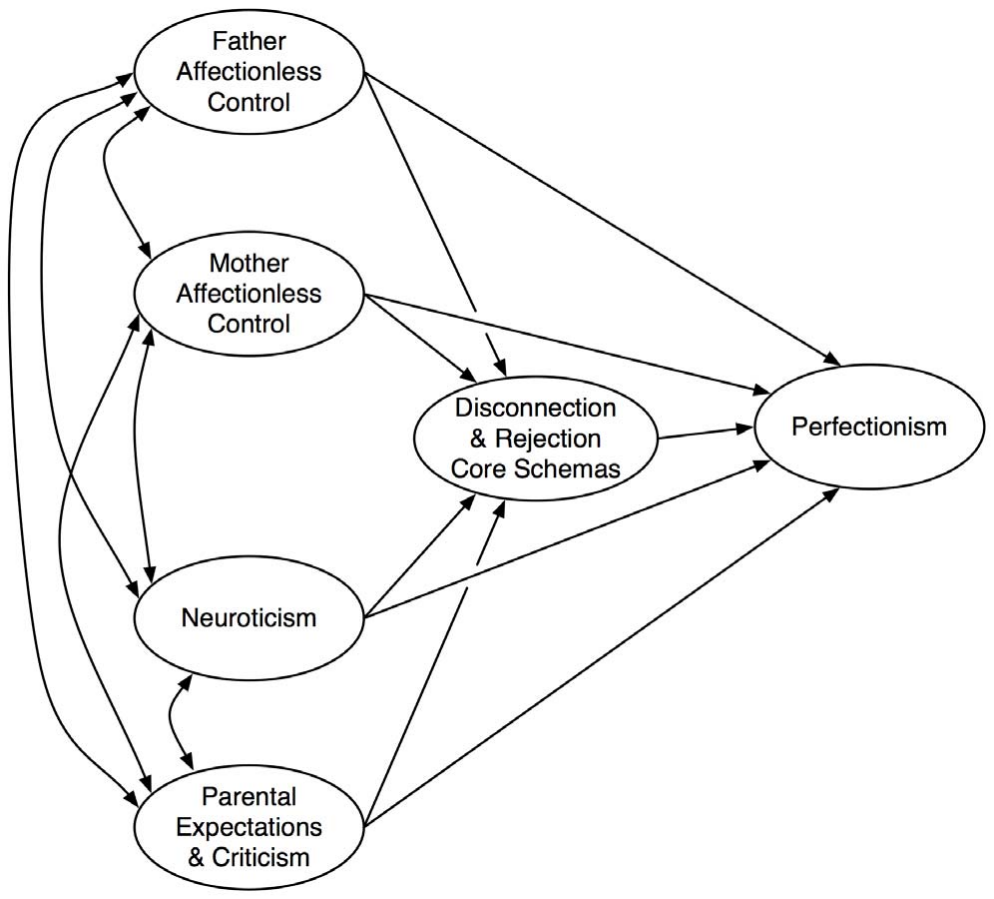
Proposed Etiological Model.

The present study aimed to develop and test an etiological model of perfectionism based on the salient etiological factors identified above. The model is depicted in Figure 1.

## Method

### Ethics Statement

This research was approved by the Curtin University Human Research Ethics Committee. Written informed consent was provided by the participants in this study, and the consent procedure was approved by the ethics committee.

### Participants

Of the 311 participants, 304 were undergoing psychological or psychiatric treatment at private practices. The remaining seven participants were undergoing psychological treatment at a university service. The practitioners reported that 90% of these clients had been referred under the Australian Medicare system that requires a diagnosis of one or more psychological disorders consistent with the DSM-IV [33].

Participants had a mean age of 36.35 (*SD* = 12.59), 74.63% were female, and 56.54% were employed. The participants attended an average of 39.74 (*SD* = 73.03) sessions with their current therapist and 57.56% had previously attended therapy.

### Procedure

The first author approached clinical psychologists and consultant psychiatrists and asked them to have their receptionists invite clients to participate and return the questionnaires either to the receptionist or the researcher via the mail. Participants were informed that their clinician would not know whether they completed the questionnaire. Participants were given a $2 scratch and win lottery ticket as a token gesture of appreciation of their time and effort. There were 800 questionnaires distributed, with 311 returned, providing a response rate of 40%.

### Measures

#### Parental Bonding Instrument (PBI) [9]

The PBI is a reliable and valid measure of one's experience of parenting [11]. Parenting styles are classified into four groups [34]: Affectionless Control (low care and high protection); Affectionate Constraint (high care and high protection); Optimal Parenting (high care and low protection); and Neglectful Parenting (low care and low protection).

#### Neuroticism Extroversion Openness to Experience-Five Factor Inventory (NEO-FFI) [35]

The Neuroticism sub-scale of the NEO-FFI measures the degree to which an individual is prone to experiencing negative emotional states and has good reliability and validity [35].

#### Frost Multidimensional Perfectionism Scale (FMPS) [12]

Perfectionism was measured by using a composite of the subscales of Concern over Mistakes, Doubts about Actions and Personal Standards as these have been suggested to represent the core criteria of perfectionism [36]. The Parental Expectations and Parental Criticism subscales of the measure were considered separately in the model. The FMPS has good reliability and validity [37].

#### Young Schema Questionnaire-Short Form (YSQ-SF) [31]

The YSQ-SF is a 75-item short version of the YSQ [38] which measures the extent to which early maladaptive schemas are present and has acceptable reliability and validity [39]. Given that the higher order domains of the YSQ-SF identified by Young [40] have received inconsistent support, however, the present research will test whether the five subscales of interest - Abandonment/Instability, Emotional Deprivation, Defectiveness/Shame, Mistrust/Abuse and Social Isolation/Alienation - form a higher order factor of Disconnection and Rejection Core Schemas.

## Results

The correlations among the study variables are presented in Table 1. Structural equation modelling (SEM) via LISREL (Version 8.540) [41] was used to test the model in Figure 1. The analyses comprised four stages. Stage 1 consisted of a CFA of each scale to determine whether a multifactor of one-factor solution provided the better fit. The results from Stage 1 were used to formulate the measurement component of the etiological model of perfectionism. At Stage 2, the measurement model was tested. If the measurement model provided an adequate fit for the data, the analysis moved to a third stage in which the structural model (Figure 1) was tested. At Stage 4, the competing structural models were compared to determine which one provided the best fit.

**Table 1:** Correlations Among the Study Variables.

	YSQ1	YSQ2	YSQ3	YSQ4	YSQ5	MPS1	MPS2	MPS3	MPS4	MPS5	NEO	MotherAC	FatherAC
YSQ1	1												
YSQ2	0.31	1											
YSQ3	0.414	0.489	1										
YSQ4	0.398	0.417	0.606	1									
YSQ5	0.416	0.575	0.574	0.671	1								
MPS1	0.108	0.173	0.087	0.102	0.166	1							
MPS2	0.243	0.401	0.344	0.393	0.524	0.603	1						
MPS3	0.193	0.308	0.386	0.462	0.461	0.305	0.581	1					
MPS4	0.166	0.176	0.152	0.176	0.192	0.308	0.368	0.262	1				
MPS5	0.392	0.244	0.314	0.32	0.316	0.252	0.448	0.359	0.678	1			
NEO	0.258	0.488	0.402	0.497	0.562	0.16	0.539	0.499	0.283	0.378	1		
MotherAC	0.464	0.266	0.335	0.272	0.288	0.159	0.345	0.236	0.376	0.528	0.278	1	
FatherAC	0.355	0.309	0.323	0.298	0.351	0.129	0.264	0.23	0.318	0.528	0.296	0.454	1

The present data were collected from six groups, each group being treated by a different group of psychologists/psychiatrists thereby creating the potential for intra-group dependencies. Given that the intra-class correlation values for the observed variables were small (mean  = .03), it was concluded that intra-group dependency would not distort the SEM analyses.

Multivariate normality was violated at all four stages. Model fit was therefore tested with the Satorra-Bentler chi-square (*χ_sb_^2^*), which corrects for non-normality [42]. Several other fit indices were used: The Root Mean Square Error of Approximation (RMSEA; values <.08 =  acceptable fit); the Non-normed Fit Index (NNFI; values >.9 =  acceptable fit); the Comparative Fit Index (CFI; values >.9 =  acceptable fit), and the Standardised Root Mean Square Residual (SRMR; values <.1 =  acceptable fit) [43].

### Descriptive statistics

The means and standards deviations for each scale are presented in Table 2.

**Table 2:** Means and Standard Deviations for the Subscales of the PBI, NEO-FFI, YSQ-SF and FMPS.

Measure	*Mean*	*SD*
PBI Father Care	17.14	9.05
PBI Father Protection	16.85	8.29
PBI Mother Care	21.21	9.94
PBI Mother Protection	21.21	9.94
NEO-FFI Neuroticism	31.94	8.88
YSQ-SF Emotional Deprivation	3.32	1.54
YSQ-SF Abandonment/Instability	3.09	1.51
YSQ-SF Mistrust/Abuse	3.06	1.47
YSQ-SF Defectiveness/Shame	3.43	1.46
YSQ-SF Social Isolation/Alienation	2.82	1.49
FMPS Personal Standards	24.97	5.37
FMPS Concern over Mistakes	28.35	8.18
FMPS Doubts about Actions	13.05	3.58
FMPS Parental Expectations	15.37	4.99
FMPS Parental Criticism	12.27	4.47

Note: PBI  =  Parental Bonding Inventory [9]; NEO-FFI  =  Neuroticism Extraversion Openness to Experience Five Factor Inventory [35]; YSQ-SF  =  Young Schema Inventory – SF [40]; FMPS  =  Frost Multidimensional Perfectionism Scale [12].

### Stage 1 SEM analysis: Confirmatory factor analyses of the questionnaires

A CFA was conducted on each of the five measures listed in Table 3. For the Neuroticism scale a one-factor solution was the only plausible solution and provided a good fit for the data. Multidimensional solutions were found for the remaining measures. The YSQ-SF multidimensional solution consisted of five factors (Defectiveness/Shame, Social Isolation/Alienation, Mistrust/Abuse, Abandonment/Instability, and Emotional Deprivation). It has been argued that these five factors are generated by a higher-order construct of Disconnection and Rejection Core Schemas and were therefore incorporated into the measurement model as separate indicators of this construct. The FMPS was found to have, a five-factor solution (PS, CM, DA, PE, PC) and a higher-order factor solution that fit equally well. In the higher-order solution, there was one higher order factor (Parental Expectations and Criticism) driving the two lower-order factors (PE and PC), and a second higher-order factor (Perfectionism) driving the three lower order factors (CM, PS, and DA). The two higher order factors were therefore studied as separate constructs in the current model.

**Table 3:** Goodness-of-fit Statistics for Confirmatory Analyses of the PBI, FMPS, YS-SF and NEO-FFI.

Measure	*χ_sb_^2^*	*df*	CFI	NNFI	SRMR	RMSEA
PBI mother (25 items): 1-Factor	2980.541	275	.866	.854	.148	.246
2-Factors: Protection, Care	2348.961	274	.918	.910	.107	.159
PBI father (25 items): 1-Factor	2750.262	275	.835	.820	.158	.234
PBI father (25 items): 2-Factors: Protection, Care	1923.716	274	.894	.884	.122	.142
FMPS (35 items): 1-Factor	3577.954	377	.827	.814	.144	.208
FMPS (35 items): 5-Factors: PE, PC, PS, DA, CM	1565.930	367	.934	.927	.093	.105
FMPS (35 items): 2 higher order factors (PEC & Perfectionism) tapping into 5 lower order factors (PE, PC, CM, DA, PS)	1620.696	371	.932	.926	.096	.106
YSQ (25 items): 1-Factor	4522.483	275	.812	.795	.142	.267
YSQ (25 items): 5-Factors: Defectiveness/Shame, Social Isolation/Alienation, Mistrust/Abuse, Abandonment/Instability, Emotional Deprivation	1116.241	265	.963	.958	.068	.104
NEO (12 items): 1-factor	328.966	54	.914	.895	.069	.130

Note. PBI  =  Parental Bonding Inventory [9], NEO-FFI  =  Neuroticism Extraversion Openness to Experience Five Factor Inventory [35], FMPS  =  Frost Multidimensional Perfectionism Scale [12], YSQ-SF  =  Young Schema Inventory – Short Form [40].

The PBI consisted of a Protection and a Care factor, which were used to partition participants into four categories [34]: Affectionless Control, Affectionate Constraint, Optimal Parenting, and Neglectful Parenting. Half the sample were in the Affectionless Control category (53.7% reported that their mothers belonged to this category, and 56.9% reported their fathers belonged to this category) suggesting that the most statistically powerful contrast among the PBI categories is between Affectionless Control and the other categories combined. The four PBI categories were recoded as a dichotomy in which Affectionless Control was coded ‘1’ and the other categories were coded ‘0’. Therefore, the constructs of Mother Affectionless Control and Father Affectionless Control were each measured by a dichotomous variable in which ‘1’ represented Affectionless Control and ‘0’ represented alternative parenting styles.

### Stage 2 SEM analysis: Testing the measurement model

**Figure 2:**
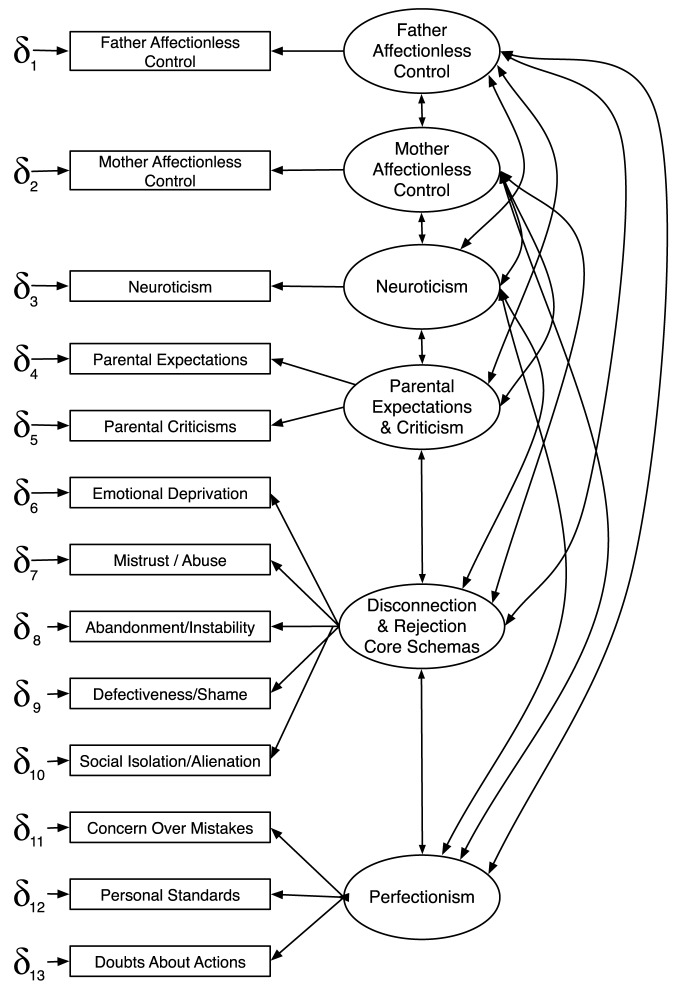
The measurement model.

The Stage 1 CFAs yielded six latent variables and 13 associated indicators for the final measurement model depicted in Figure 2. Because Neuroticism, Mother Affectionless Control, and Father Affectionless Control are all one-indicator constructs, their measurement errors could not be estimated from the data and therefore had to be estimated from the reliabilities of their respective indicators.

The measurement model provided a good fit to the data. The CFI and NNFI were .956 and .934 respectively (>.9), the SRMR was .072 (<.1), and the RMSEA was .095 (with a 90% CI encompassing the cutoff of .08). Moreover, using Hair et al. 's [44] formula, and a cutoff of .7, the three latent variables with multiple indicators showed good construct validity (Parental Expectations and Criticisms  = .83, Disconnection and Rejection  = .84, and Perfectionism  = .78).

### Stage 3 SEM analysis: Testing the structural model

The structural model provided a good fit to the data. The CFI and NNFI were .955 and .934 respectively (>.9), the SRMR was .072 (<.1), and the RMSEA was .095 (with a 90% CI encompassing the cutoff of .08). The path coefficients for each of the pathways in the structural model, and their significance, are presented in Table 4.Three of the pathways in the saturated 9-pathway model were non-significant and were dropped from the model.

**Table 4:** Path Coefficients for the Nine-Pathway Structural Model.

*Pathway*	*Path Coefficient (Standard Error)*	*z-value*	*p-value*
Father Affectionless Control→ Disconnection & Rejection Core Schemas	.211 (.078)	2.717	.007**
Mother Affectionless Control → Disconnection & Rejection Core Schemas	.149 (.073)	2.051	.040*
Parental Expectations & Criticism → Disconnection & Rejection Core Schemas	−.062 (.072)	−0.858	.391
Neuroticism → Disconnection & Rejection Core Schemas	.621 (.088)	7.019	.000***
Disconnection & Rejection → Perfectionism	.232 (.095)	2.447	.014*
Father Affectionless Control → Perfectionism	−.143 (.075)	−1.907	.057
Mother Affectionless Control → Perfectionism	.090 (.069)	1.305	.192
Parental Expectations & Criticism → Perfectionism	.239 (.075)	3.207	.001**
Neuroticism → Perfectionism	.359 (.094)	3.824	.000***

Note. *p<.05; **p<.01; *** *p*<.01.

### Stage 4 SEM analysis: Comparing the nine-pathway and the six-pathway structural models

The fit statistics for the reduced six-pathway model were CFI  = .954 (>.9), NNFI  = .937 (>.9), SRMR  = .072 (<.1), and RMSEA  = .093 (with a 90% CI encompassing the cutoff of .08) showing it fit the data reasonably well. The chi-square different test comparing the 6- and 9-pathway models was non-significant (χ^2^ (56) = 205.94; χ^2^ (53) = 202.14; χ^2^diff (3) = 3.80, *p* = .284) indicating that both models fit the data equally well. The more parsimonious 6-pathway model is therefore preferred. The path coefficients for the 6-pathway model are reported in Table 5. Standardised path estimates and standard errors for each of the three indirect pathways passing through Disconnection and Rejection were estimated with a bootstrapping procedure based on 1000 draws as implemented by Mplus (Version 5.2) [46]. The bootstrapped path estimate and standard error for the indirect pathway from Father Affectionless Control to Perfectionism were .095 and .038 respectively (*z* = 2.50, *p* = .012); from Mother Affectionless Control to Perfectionism, the parameters were .087 and.040 respectively (*z* = 2.18, *p* = .030); and from Neuroticism to Perfectionism, the parameters were .019 and .006 respectively (*z* = 3.17, *p* = .002). All three indirect pathways were therefore statistically significant.

**Table 5:** Path Coefficients for the Six-Pathway Structural Model.

Pathway	Path Coefficient (Standard Error)	z-value	p-value
Father Affectionless Control → Disconnection & Rejection Core Schemas	.192 (.071)	2.688	.007**
Mother Affectionless Control → Disconnection & Rejection Core Schemas	.123 (.058)	2.121	.034*
Neuroticism → Disconnection & Rejection Core Schemas	.608 (.086)	7.095	.000***
Disconnection & Rejection Core Schemas → Perfectionism	.204 (.087)	2.332	.020*
Parental Expectation & Criticism → Perfectionism	.212 (.057)	3.715	.000***
Neuroticism → Perfectionism	.371 (.092)	4.024	.000***

Note. *p<.05; **p<.01; ***p<.001.

**Figure 3:**
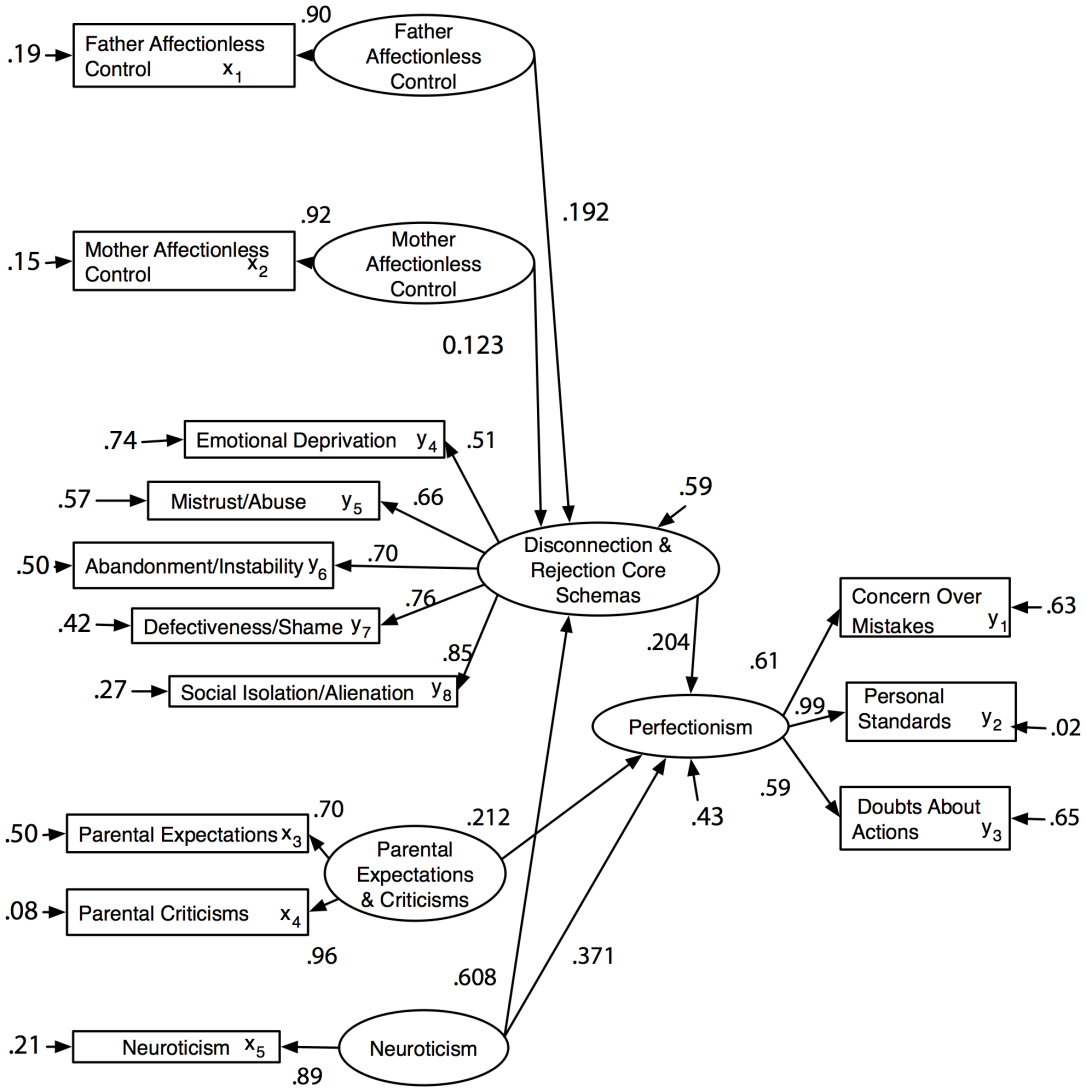
The measurement and structural components of the final six-pathway model.

The measurement and structural components for the six-pathway model are depicted in Figure 3. This represents the final etiological model of perfectionism.

The correlations among the latent variables as seen in Table 6 all reached significance (*p*<.001). The 6-pathway model can be used to explain the correlations between Perfectionism and the other latent variables. First, the relationship between Father Affectionless Control and Perfectionism (*r*(309) = .293, *p*<.001) and between Mother Affectionless Control and Perfectionism (*r*(309) = .374, *p*<.001) are both mediated by Disconnection and Rejection. In other words, Affectionless Control did not have a direct impact on perfectionism; it had an indirect impact via Disconnection and Rejection. Second, the relationship between Neuroticism and Perfectionism (*r*(309) = .608, *p*<.001) arises from Neuroticism having both a direct and indirect impact (via Disconnection and Rejection) on perfectionism. Third, the relationship between Parental Expectations and Criticism and Perfectionism (*r*(309) = .460, *p*<.001) is *not* mediated by Disconnection and Rejection. Thus, Parental Expectations and Criticism has a direct impact on Perfectionism. Finally, the relationship between Disconnection and Rejection schemas and Perfectionism (*r*(309) = .561, *p*<.001) reflects the direct impact of Disconnection and Rejection on Perfectionism as well as the impact of each of the four variables on both Disconnection and Rejection and Perfectionism.

**Table 6:** Correlation Matrix of Latent Variables.

	DRCS	Perfectionism	MAC	FAC	Neuroticism	PEC
DRCS	1.000					
Perfectionism	.561	1.000				
MAC	.438	.374	1.000			
FAC	.485	.293	.548	1.000		
Neuroticism	.722	.608	.339	.370	1.000	
PEC	.420	.460	.584	.595	.434	1.000

Note. p<.001 for all correlations; MAC  =  Mother Affectionless Control and FAC  =  Father Affectionless Control of the Parental Bonding Inventory [9]; PEC  =  Parental Expectations and Criticisms of the Frost Multidimensional Perfectionism Scale [12]; DRCS  =  Disconnection & Rejection Core Schemas of the Young Schema Questionnaire – Short Form [40]; Neuroticism scale of the Neuroticism Extraversion Openness to Experience Five Factor Inventory [35].

## Discussion

The aim of this study was to propose and test an etiological model of perfectionism. Consistent with previous etiological models that have not been tested [4], parental factors were found to be pertinent in the development of perfectionism with a direct relationship between parental expectations and criticism, and perfectionism and an indirect relationship between parental bonding and perfectionism that was mediated by core schemas. Furthermore, the role of a generalized disposition towards negative affect in the development of perfectionism, important in etiological models of anxiety [6], was supported by the finding that Neuroticism had both an indirect relationship, mediated by core schemas, and a direct relationship with perfectionism. The current research provided partial support for Flett et al. 's [4] etiological model with parental expectations and criticism being directly related to perfectionism. Furthermore, Flett et al. theorized that a childhood temperament, characterized by high levels of emotionality, is an etiological factor in the development of perfectionism. The present findings of a relationship between neuroticism and perfectionism are consistent with this suggestion. While, Flett et al. did not specifically mention neuroticism in their model, clearly this construct has conceptual similarities to high levels of emotionality. It would be useful for future research to compare the present model with Flett et al. 's model in a clinical population. Further research is also needed to examine the mediating relationships between the etiological factors proposed in Flett et al. 's model. Our findings support the role of critical parenting, core schemas and neuroticism as important components leading to the development of perfectionism, as discussed below.

The different relationships between the two parenting factors and perfectionism are intriguing. There was a direct, but not an indirect relationship, between parental expectations and criticisms and perfectionism. In contrast, there was only an indirect relationship between affectionless control and perfectionism mediated by disconnection and rejection core schemas. One hypothesis for this may be that as parental expectations and criticism represent explicit verbal comments, this may link directly to the development of perfectionism. For example, hearing from a parent “I expect nothing less than straight A's”, may be sufficiently explicit to contribute directly to the development of perfectionism. As affectionless control represents an implicit experience of parental bonding, a person may need to develop an internal belief regarding their experience. For example, experiencing low levels of care and high levels of protection from parents may cause a person to draw inferences about what this means about their self-concept “e.g., If I get straight A's, then I will get the love from my parents I desire”. This hypothesis is consistent with theoretical accounts that core beliefs develop as a way of making sense of our thoughts, feelings and behaviors in response to our interactions with others; for example: [21].

Neuroticism was found to have both a direct and indirect (via disconnection and rejection core schemas) relationship with perfectionism mediated by disconnection and rejection core schemas. The results contribute to the wealth of data that shows a relationship between neuroticism and perfectionism; for example [16].

The finding that disconnection and rejection core schemas are associated with perfectionism are consistent with studies that have consistently found a relationship between perfectionism and cognitions [27], [45], [29]. Furthermore, our findings that perfectionism is related to the schema domain of disconnection is the same as found by Boone et al [32]. The present findings support the theories of Beck [21] and Young [40], when viewed in the context of the perfectionism literature, in that, disconnection and rejection schemas develop as a result of personality and parenting factors, and subsequently perfectionism develops as an intermediate belief that serves as a contingent function that strengthens the core schemas.

### Clinical Implications

It would be useful for further research to determine if cognitive-behavioural treatment of perfectionism; for example [3] results in reductions of the core schemas that were identified as important in this research. If schemas are not found to reduce as a product of CBT for perfectionism [3] then this may suggest it is important for future research to examine if targeting core schema such as disconnection and rejection that have been found in our study and others; for example [32] to be significantly related to perfectionism, helps to increase the efficacy of treatments for perfectionism. We recommend that clinicians assess for the schema domains of disconnection and rejection, in their development of a formulation of perfectionism in order to include these schema as potentially important in understanding the development of their client's perfectionism. Furthermore, in an individualized formulation of perfectionism it may also be useful to include parental expectations and criticism and a generalized disposition towards the experience of negative emotions as predisposing factors in order to help the client understand the development of their perfectionism.

### Limitations

The main limitation was that the diagnosis of the participants was not determined. Clinicians could not be queried regarding client diagnosis because, for ethical reasons, they were not allowed to know that their clients were participants. Therefore, the results of this study are limited in their generalizability as the diagnosis of the sample was unknown and it was only clear that they were in treatment for psychological problems either through a psychiatrist or clinical psychologist. Not knowing the diagnosis of the sample is a significant limitation that limits the generalizability of the findings. Despite this, the private practitioners reported that 90% of their clients had been referred on the basis that they met at least one DSM-IV [33] diagnosis. Furthermore, SEM can only determine whether our cross-sectional data are consistent with our proposed causal model, it cannot prove cause-and-effect relationships. Our results, therefore, cannot be taken as evidence that parenting factors, schemas of disconnection and rejection and neuroticism cause perfectionism. Further research utilizing a longitudinal design is required to bolster the causal hypothesis.

## Conclusions

The strength of the current study is that it is the first to date to empirically test an etiological model of perfectionism. Understanding the etiology of perfectionism is important as given it is a risk factor for the development of disorders (e.g., eating disorders) [1] and a known pertinent maintaining factor of numerous disorders [1] then understanding etiology has important implications for the prevention of perfectionism. Future research should determine if it is possible to intervene with parental factors, core schema and neuroticism variables in order to prevent perfectionism and the subsequent influence of this variable on the development of a wide range of psychopathology.
